# Delivering Oxidation Resistance-1 (OXR1) to Mouse Kidney by Genetic Modified Mesenchymal Stem Cells Exhibited Enhanced Protection against Nephrotoxic Serum Induced Renal Injury and Lupus Nephritis

**DOI:** 10.4172/2157-7633.1000231

**Published:** 2014-09-10

**Authors:** Yajuan Li, Wei Li, Chu Liu, Mei Yan, Indu Raman, Yong Du, Xiangdong Fang, Xin J. Zhou, Chandra Mohan, Quan-Zhen Li

**Affiliations:** 1 Key Laboratory of Medical Genetics, Wenzhou Medical University School of Laboratory Medicine & Life Science, Wenzhou, 325035, China; 2 Laboratory of Disease Genomics and Individualized Medicine, Beijing Institute of Genomics, Chinese Academy of Sciences, Beijing, 100029, China; 3 Department of Biomedical Engineering, University of Houston, Houston, TX, 77204, USA; 4 Renal Path Diagnostics, Pathologist BioMedical Laboratories, Lewisville, TX, 75067, USA; 5 Department of Immunology and Internal Medicine, University of Texas Southwestern Medical Center, Dallas, TX, 75390, USA

**Keywords:** Oxidation resistance 1, lupus nephritis, mesenchymal stem cell, oxidative stress, inflammation, apoptosis

## Abstract

**Objective:**

To elucidate the role of oxidation resistance 1 (OXR1) gene. Oxidative stress plays a pivotal role in pathogenesis of immune-mediated nephritis. Recently we identified oxidation resistance 1 (OXR1) is conventionally expressed in eukaryotes and has an ability to prevent oxidative damage caused by various oxidative stresses. However the protective effect of OXR1 in immune-associated inflammatory response and oxidative damage is not clear and will be investigated in this study.

**Methods:**

We utilized mesenchymal stem cells (MSCs) as vehicles to carry OXR1 into the injured kidneys of nephritis model mice and investigated the influence of *OXR1* on glomerulonephritis. Human *OXR1* gene was integrated into genome of MSCs via lentiviral vector, and established hOXR1-MSC cell line which still maintains the differentiation property. *129/svj* mice with anti-glomerular basement membrane (GBM) challenge and spontaneous lupus mice *B6.Sle1.Sle2.Sle3* were injected with hOXR1-MSCs (*i.v.* injection) to evaluate the function of hOXR1. Immunohistochemistry was used to appraise the renal pathology and Tunel staining was applied to detect cell apoptosis.

**Results:**

Compared with control mice, hOXR1-MSCs administration showed significantly decreased blood urea nitrogen (BUN), proteinuria and ameliorated renal pathological damage. hOXR1-MSCs transplantation significantly reduced macrophage and T lymphocyte infiltration by inhibiting the expression of CCL2, CCL7, IL-1β, IL-6 and NFκB in mouse kidney. Moreover, hOXR1-MSCs prevented hydrogen peroxide (H_2_O_2_)-induced oxidative stress and its implantation reduced nitric oxide (NO) in mouse serum and urine to inhibit tubular cell apoptosis.

**Conclusion:**

OXR1-MSCs transplantation may exert a certain protective effect on nephritis by suppressing inflammation and oxidative stress.

## Introduction

Lupus Nephritis (LN) is a leading cause of morbidity and mortality in Systemic lupus erythematosus (SLE) [1,2], and oxidative stress is a major contributor to disease in LN [3]. The pathogenesis of LN involves a complicated interaction of multiple factors, the imbalance between oxidative stress and anti-oxidative chemokines is considered to be a universal factor involved in the manifestation of various clinical features in SLE patients [3]. The infiltration of various leucocytes into the inflamed kidneys plays a critical role in the pathogenesis of LN partly because they produce reactive oxygen species (ROS) at the sites of inflammation. Excessive ROS disturb cell redox status, damage macromolecules and can modulate the expression of a variety of immune and inflammatory molecules which consequently exacerbate inflammation and aggravate tissue damage [4]. Therefore, studying the complex interplay of oxidative stress and anti-oxidants may deepen our understanding of the disease, and possibly lead to novel therapeutics.

We had previously reported that some strains of mice (such as DBA/1, 129/svj, NZW) are susceptible to anti-GBM nephritis while other mouse strains (such as B6, BALB/c) are not [5,6]. A microarray analysis of the renal cortex from these 5 strains following anti-GBM disease challenge indicated several disease associated or disease-protective genes [6,7]. This study focuses on one of those genes because of its anti-oxidant role. We found that the anti-oxidant gene, oxidation resistance 1 gene (*OXR1*) was significantly up-regulated in kidneys of nephritis resistant mice (*B6*, *BALB/c*) compared with the sensitive mice (*DAB1*, *129/svj, NZW*) [8]. Thus, the increased expression of *OXR1* was correlated with the milder inflammation noted in kidney of anti-GBM challenged B6 and BALB/c mice.

*OXR1* is widely expressed in various eukaryotes. It participates in the detoxification of ROS and exhibits an important role in protecting yeast and human cells against oxidative damage [5]. *OXR1* as a free radical scavenger plays a protective role in the early stage of hydrogen peroxide or hyperoxia-induced death [9–11]. *OXR1* suppresses oxidative DNA damage in *Escherichia coli* and reduces the accumulation of mutations in the bacterial genome [12]. OXR1 exerts a protective effect against oxidative stress either directly or by functioning as a cofactor to modulate transcriptional activation of catalase (CAT) and glutathione peroxidase (GPX) [13]. OXR1 also protects neuronal cells against oxidative stress and it may be important in neurodegenerative diseases [14]. Although *OXR1* protects cells from oxidative DNA damage and its protective effect against oxidative DNA damage are inducible upon exposure to oxidative stress [15], its molecular mechanism remains to be defined.

Oxidative stress caused by poor detoxification of ROS may play a role in the development of SLE and increased oxidative stress may be important in glomerular injury [16–18]. Hence, in this study, we investigated the impact of OXR1 on suppressing oxidative damage in experimentally induced anti-GBM nephritis, and spontaneously arising lupus nephritis.

## Materials and Methods

### Human subject and animal study declaration

All human kidney sections derived from healthy subjects and SLE patients with informed consent. The use of human kidney specimens was approved by local ethics committees. All animal experiments were performed according to the guidelines of University of Texas Southwestern Medical Center Institutional Animal Care and Use Committee and were approved by the local authorities.

### Bone marrow mesenchymal stem cell isolation and culture

Mesenchymal stem cells (MSCs) were isolated from bone marrow (BM) of 2 month old female *B6* mice. MSCs were seeded in cell culture flask containing Dulbecco’s modified Eagle’s medium (DMEM) plus 10% FBS and penicillin (100 U/ml)-streptomycin (0.1 mg/ml) (Invitrogen, Carlsbad, CA) and cultured in 5% CO_2_ incubator at 37°C. The cultured cells were replenished with fresh medium every three days. MSCs in passage 2 were identified using six markers (CD11b, CD29, CD34, CD44, CD45 and Sca-1) with flow cytometric analysis. CD29, CD44 and Sca-1 are positive immunostaining markers, while CD11b, CD34 and CD45 are negative immunostaining markers for MSCs. Only cells from lower passage numbers (less than 10 passages) were used for the experiments in this study.

### Establishing stable MSC cell line with constitutive expression of human *OXR1*

The Human *OXR1* gene coding region was amplified by polymerase chain reaction (PCR) with forward primer (5′GC*TCTAGA*GGCCCCAGTTCCGCGTC3′) and reverse primer (5′GC*GGATCC*AGAGAGCATTTTATTTA3′) using IMAGE CLONE 5496492 (Source BioScience, Nottingham, UK). XbaI and BamHI were used to clone the product into pCDH lentiviral vector (System Biosciences, Mountain view, CA). The recombinant pCDH-hOXR1 and packaging plasmid mixture were transfected into 293TN packaging cells and the viral particles were collected after 48 hr of cell transfection. Cultured MSCs were infected with pCDH-hOXR1 virus and 8 μg/ml polybrene (Sigma, St Louis, MO) for 72 hr, until the cells were 50–60% confluent, they were sorted with flow cytometry using the GFP marker. Expression of recombinant hOXR1 in MSCs was identified by quantitative real time polymerase chain reaction (QPCR) and western blot. MSCs infected with pCDH vector were used as control.

### Detection of apoptotic cells caused by hydrogen peroxide

The three MSC cell lines (MSCs, pCDH-MSCs and hOXR1- MSCs) were seeded in 6-well plates. When the cells reached 60–70% confluence, they were treated with 0.5 mM hydrogen peroxide (H_2_O_2_) for 6 hr. The cells were washed with phosphate-buffered saline (PBS) twice, and fixed with 4% paraformaldehyde (PFA) for 25 min and washed with PBS twice. Apoptotic cells were measured using an in situ cell death detection kit (Roche, Indianapolis, IN). The procedure was performed according to manufacturer’s protocol. Finally, stained cells were enumerated under the microscope.

### Anti-GBM induced nephritis mouse model

Two month old female *129X1/svj* mice purchased from Jackson Lab (Bar Harbor, ME, USA) were used to study the impact of hOXR1-MSCs on anti-GBM induced nephritis. All mice were housed at a constant room temperature and humidity and had free access to drinking water and food. 12 mice were randomly divided into 2 groups of 6 mice per group. All mice were subjected to anti-GBM disease as described previously [5]. After anti-GBM challenge for 2 days, the mice in Group 1 were injected with 10^6^ hOXR1-MSCs via tail vein, and the mice in Group 2 were injected with the same numbers of pCDH-MSCs. Blood and urine samples were collected from each mouse at day 0 (pretreatment), day 14 and day 21 after anti-GBM challenge. Experimental mice were sacrificed on day 21 and tissues were collected and frozen at −80°C for further analysis. Kidneys were removed and fixed in formaldehyde for immunohistochemical analysis.

### Spontaneous lupus nephritis mouse model

Six month old female *B6.Sle1.Sle2.Sle3* mice were used to test the therapeutic effect of hOXR1-MSCs on lupus nephritis. Ten mice were divided into two groups of 5 mice per group. One group of mice was injected with 10^6^ hOXR1-MSCs and another group of mice were injected with the same numbers of pCDH-MSCs as controls. All mice were injected twice, four weeks apart, and observed for a period of 8 weeks. Blood and urine samples were collected every four weeks after injection to measure blood urea nitrogen (BUN) and proteinuria. All mice were sacrificed 8 weeks after injection and kidneys were removed and snap frozen in liquid nitrogen and fixed in formaldehyde for pathological examination.

### Measurement of proteinuria and BUN

Twenty-four-hour urine samples were collected from all experimental mice. Urinary protein concentration was measured with Coomassie Plus protein assay kit (Thermal scientific, Rockford, IL). The collected blood samples were centrifuged, and then the sera were collected to measure BUN with a urea nitrogen kit (Sigma-Aldrich, St. Louis, MO). All measurements were performed following the manufacturer’s instruction.

### Nitric oxide (NO) detection

Nitric oxide level in mouse serum and urine was detected with QuantiChrom Nitric Oxide Assay Kit (BioAassay Systems, Hayward, CA). The serum samples were deproteinated before NO detection by pre-treatment with ZnSO_4_ and NaOH. Then, 100 μl of serum or urine sample was mixed with 200 working reagent and incubated 150 min at room temperature. Optical density (OD) of each reaction was read at 540 nm. Nitric oxide levels were calculated based on the standard curve.

### Renal histological analysis

Kidney tissues were fixed in 10% formaldehyde, dehydrated, and paraffin embedded. Tissue sections were stained by Periodic Acid Schiff (PAS). The severity of glomerulonephritis (GN) was graded on a 0–4 scales, as previously described [5]. The severity of tubulointerstitial nephritis (TIN) was also graded on a 0–4 scale, as detailed previously [5].

### Tunel staining

The kidney sections of SLE patients with LN and controls, as well as renal sections from *B6.Sle1. Sle2.Sle3* mice treated with hOXR1-MSCs or pCDH-MSCs were dewaxed with xylene and washed with 100% ethanol, rehydrated and washed in decreasing concentration of ethanol, and then immersed in 0.85% NaCl and washed with PBS twice. Apoptosis detection on tissue sections was performed with

DeadEnd™ Colorimetric Apoptosis Detection System (Promega Corporation, Madison, WI). The slides were fixed with 4% paraformaldehyde (PFA) for 15 min and immersed in PBS twice for 10 min, then permeabilized with 20 μg/mL of Proteinase K solution and incubated for 20 min at room temperature. Then the slides were washed in PBS and fixed with 4% PFA for 5 min, washed and equilibrated with equilibration buffer for 10 min. 100 μl of TdT mix was added to the tissue section and incubated for 60 min at 37°C, and then the slides were immersed in 2XSSC for 15 min, and washed in PBS. The slides were immersed in 0.3% hydrogen peroxide for 3 min and washed in PBS. Then, 100 μl Streptavidin HRP (diluted 1:500 in PBS) were added and incubated 30 min at room temperature. After the slides were washed in PBS, 100 μl DAB was added to stain the slides. The slides were washed in deionized water, and were mounted in mounting medium. Tunel-positive apoptotic cells were enumerated under a light microscope.

### Renal immunohistochemistry

Immunohistochemical staining was performed on formalin-fixed, paraffin-embedded kidney tissue as described previously [19]. Infiltration of leukocytes within the glomeruli and interstitial regions were detected by staining with specific antibodies to T lymphocytes (anti-CD3, Abcam) and to macrophages (anti-Iba1, Abcam). In addition, the expression and location of *OXR1* in the kidneys of SLE patients with different degrees of disease (WHO class II, III, IV), as well as in mouse kidneys after hOXR1-MSCs transplantation were also detected by staining with anti-OXR1 specific antibodies (rabbit anti- OXR1, 1:2000 dilution, Sigma, St. Louis, MO).

Catalase (CAT) and glutathione peroxidase 1 (GPX1) were detected in mouse kidney sections with specific antibodies (anti-Catalase antibody, 1:500 dilution, Sigma-Alorich, St, Louis, MO; anti-GPX1 antibody, 1:300 dilution, Abcam, Cambridge, MA). iNOS expression was assayed with an anti-iNOS antibody (Abcam, Cambridge, MA, 1:200 dilution,).

The number of CD3 positive cells and Iba1 positive cells was counted in 10 high power fields (HPF). The intensity of immunohistochemical staining for CAT, GPX1 and iNOS was semi-quantitatively evaluated using the following scales as described previously [20]: 0, none; 1+, mild; 2+, moderate; and 3+, marked.

### Western blot analysis

Western blot analysis was performed with the cytosolic fraction of mouse kidney, other organs and cell lysates from cultured hOXR1- MSCs and pCDH-MSCs to detect the expression of hOXR1. GAPDH was used as an internal control. The procedure was performed as previously described [21].

### Quantitative real time polymerase chain reaction

Total RNA was extracted from kidney tissue and cell lines (hOXR1- MSCs and pCDH-MSCs) with RNeasy kit (Qiagen, Valencia, CA). RNA was transcribed to generate cDNA (Invitrogen, Carlsbad, CA). QPCR was carried out as described previously [21]. 18S rRNA was used as an internal control. The assay IDs of the genes measured are as follow: Catalase (CAT), CCL2, CCL7, CD40L, CXCL2, CXCL10, Fas1, GPX1, IL-1β, IL-6, IL-8γβ, IL-10, IL-12α, MMP2, MMP10, NFκB, OXR1, TGF-β and TNF.

### Cytokine detection on mouse serum

Mouse cytokine magnetic 20-Plex panel (Invitrogen, Carlsbad, CA) was used to assay the levels of 20 cytokines (FGF-basic, GM-CSF, IFN-γ, IL-1α, IL-1β, IL-2, IL-4, IL-5, IL-6, IL-10, IL-12, IL-13, IL-17, IP-10, KC, MCP-1, MIG, MIP-1α, TNF-α, and VEGF) in *B6.Sle1. Sle2.Sle3* mouse serum. The procedure was performed following the manufacturer’s instruction. Finally, cytokine concentrations in mouse serum were measured with Luminex^®^ xMAP^®^ system.

### Statistical analysis

Student’s t-test was used in statistical evaluation of the data as appropriate using Graphpad Prism 6. Group data were expressed as means ± SEM. Value of all parameters was considered to be significantly different at a value of p<0.05.

## Results

### Characteristics of MSCs and generation of hOXR1-MSC stable cell line

**Figure 1:**
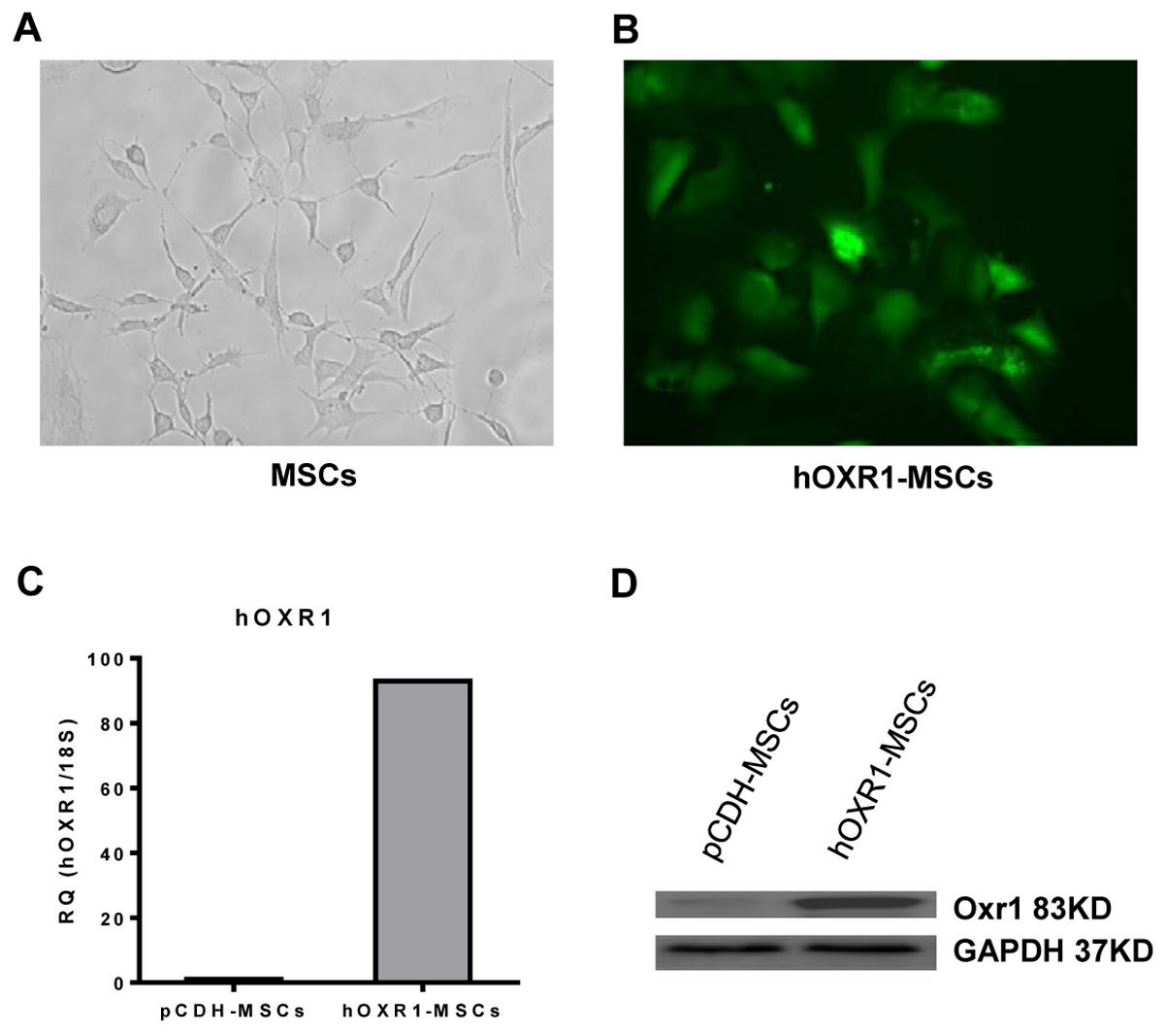
Characterization of mesenchymal stem cells and hOXR1 expression in hOXR1-MSCs. (A) Fibroblast-like spindle shape of MSCs are shown. (B) MSCs transduced with human OXR1 and GFP. (C) Human OXR1 mRNA expression level in MSCs by QPCR. (D) Human OXR1 protein expression in MSCs by western blot.

MSCs isolated from mouse bone marrow were characterized by immunocytochemical staining with six markers and were identified morphologically based on their fibroblast-like spindle shape (Figure 1A and 1B), and their differentiation potential to differentiate into osteocytes and adipocytes by culturing the MSCs in appropriate induction media for 3 weeks. The MSCs with stable hOXR1 expression were selected with puromycin resistance and confirmed by QPCR and western blot to express *OXR1* (Figure 1C and 1D).

### hOXR1-MSCs resisted H_2_O_2_-induced apoptosis

**Figure 2:**
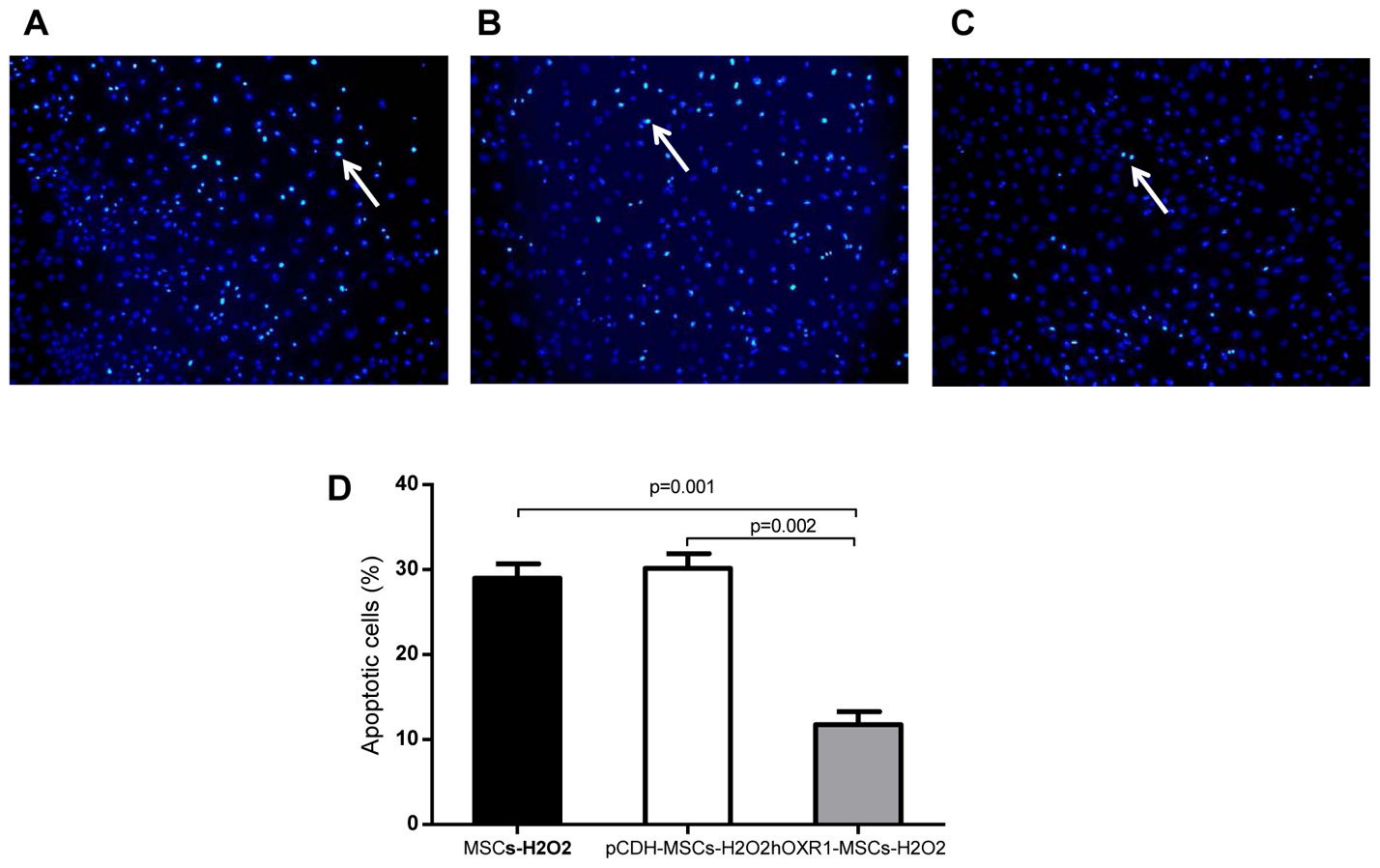
Hydrogen peroxide induced apoptosis in MSCs. (A–C) Representative tunel staining images of MSC cells after treatment with hydrogen peroxide (H_2_O_2_). (A) MSCs with H_2_O_2_ treatment for 6 hr. (B) pCDH-MSCs with H_2_O_2_ treatment for 6 hr. (C) hOXR1-MSCs with H_2_O_2_ treatment for 6 hr. (D) Quantitative analysis of apoptotic cells expressed as a percentage of total cell number (p<0.05). Each bar represents the average of 5 observations per group.

After the three cell lines (MSCs, pCDH-MCSs and hOXR1-MSCs) were treated with H_2_O_2_, apoptosis was assayed by the Tunel staining. Representative images of apoptotic cells in MSCs, pCDH-MSCs and hOXR1-MSCs are shown in Figure 2A–2C. MSCs transduced with human *OXR1* were more resistant to H_2_O_2_-induced apoptosis compared with MSCs and pCDH-MSCs. The percentage of apoptotic cell in MSCs, pCDH-MSCs and hOXR1-MSCs were 29.30 ± 0.78, 30.17 ± 0.98 and 11.73 ± 0.91% respectively. Quantitative analysis is shown in Figure 2D (p<0.05).

### *OXR1* expression in kidneys of different strain mice

**Figure 3:**
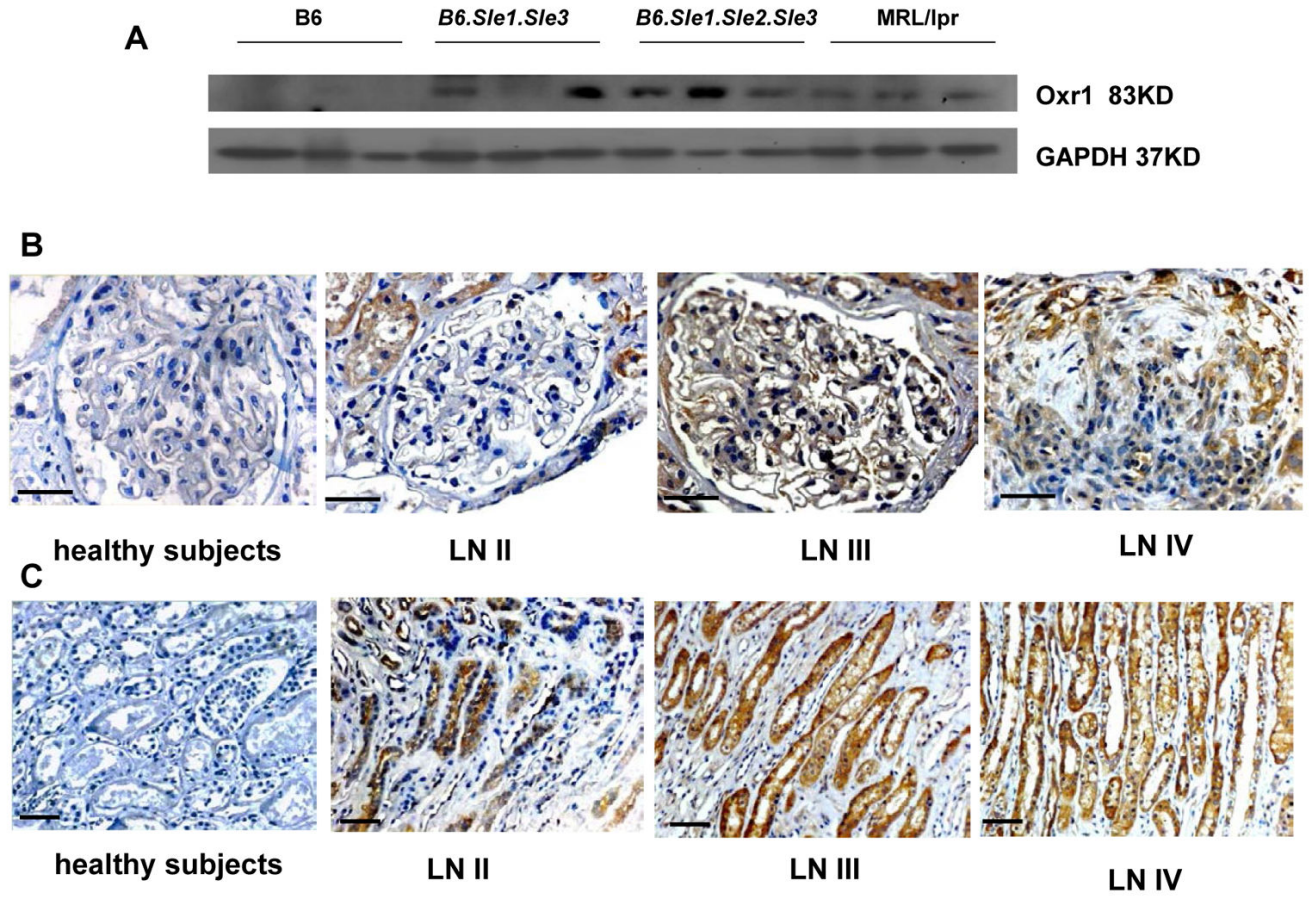
OXR1 expression in kidney in murine lupus and SLE patients with LN. OXR1 expression in *B6*, *B6.Sle1.Sle3*, *B6.Sle1.Sle2.Sle3*, and MRL/lpr. Female mice aged to 8 months. OXR1 expression in renal cortex of control subjects and SLE patients with LN II–IV. OXR1 expression in renal medulla of control subjects and SLE patients with LN II–IV. Shown are representative images of 5 samples studied for each group (upper row: original magnification 400X. lower row: original magnification 200X, scale bars=50 μm).

*OXR1* expression was detected in kidneys of different strains of mice, including *B6*, and murine model of lupus model mice (*B6.Sle1. Sle3*, *B6.Sle1.Sle2.Sle3* and MRL/lpr) by western blot. OXR1 was highly expressed in kidneys in mice with spontaneous lupus nephritis, but not in healthy *B6* mouse. The results are shown in Figure 3A.

### *hOXR1* expression in kidneys of SLE patients

Based on immunohistochemical analysis, OXR1 protein was highly expressed in renal glomerular and tubular region of WHO class II–IV LN patients, whereas there was no *OXR1* expression in disease control (minimal change disease), as shown in Figure 3B and 3C.

### *hOXR1* expression in vivo mouse after hOXR1-MSCs transplantation

**Figure 4:**
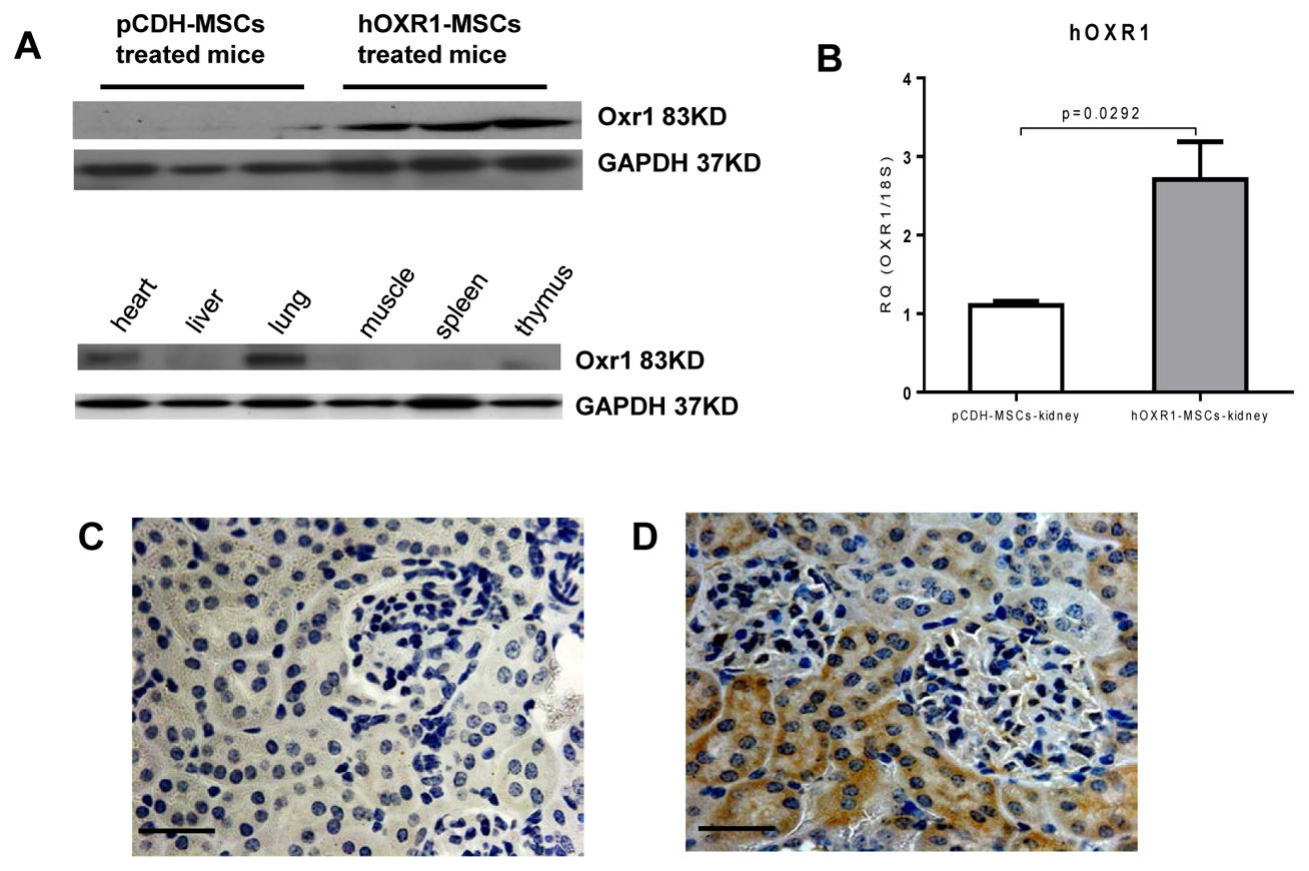
hOXR1 expression *in vivo* after hOXR1-MSC injection into the mice. (A) hOXR1 expression in kidney and other organs by western blot. (B) hOXR1 mRNA expression in kidney by QPCR (n=3 in each group). (C–D) Representative image of hOXR1 expression in mouse kidney tissue section of pCDH-MSCs treated mice (C) and hOXR1-MSCs treated mice (D). (Original magnification 400X, scale bars=50 μm). shown images are representative of 5 samples per group.

To monitor *hOXR1* expression and location in mouse organs after MSC injection, the different organs were collected for detecting *OXR1* expression by QPCR and western blot. *hOXR1* expression was detected in the kidneys of hOXR1-MSCs injected mice but not in pCDH-MSCs injected mice (Figure 4A and 4B). Among the other tissues, hOXR1 expression was only detected in the lung and heart of hOXR1-MSCs injected mice, but not in the spleen, liver, thymus and muscle (Figure 4A). IHC detection verified these findings, as shown in Figure 4C and 4D.

### hOXR1-MSCs transplantation suppressed anti-GBM induced nephritis in 129 mice

**Figure 5:**
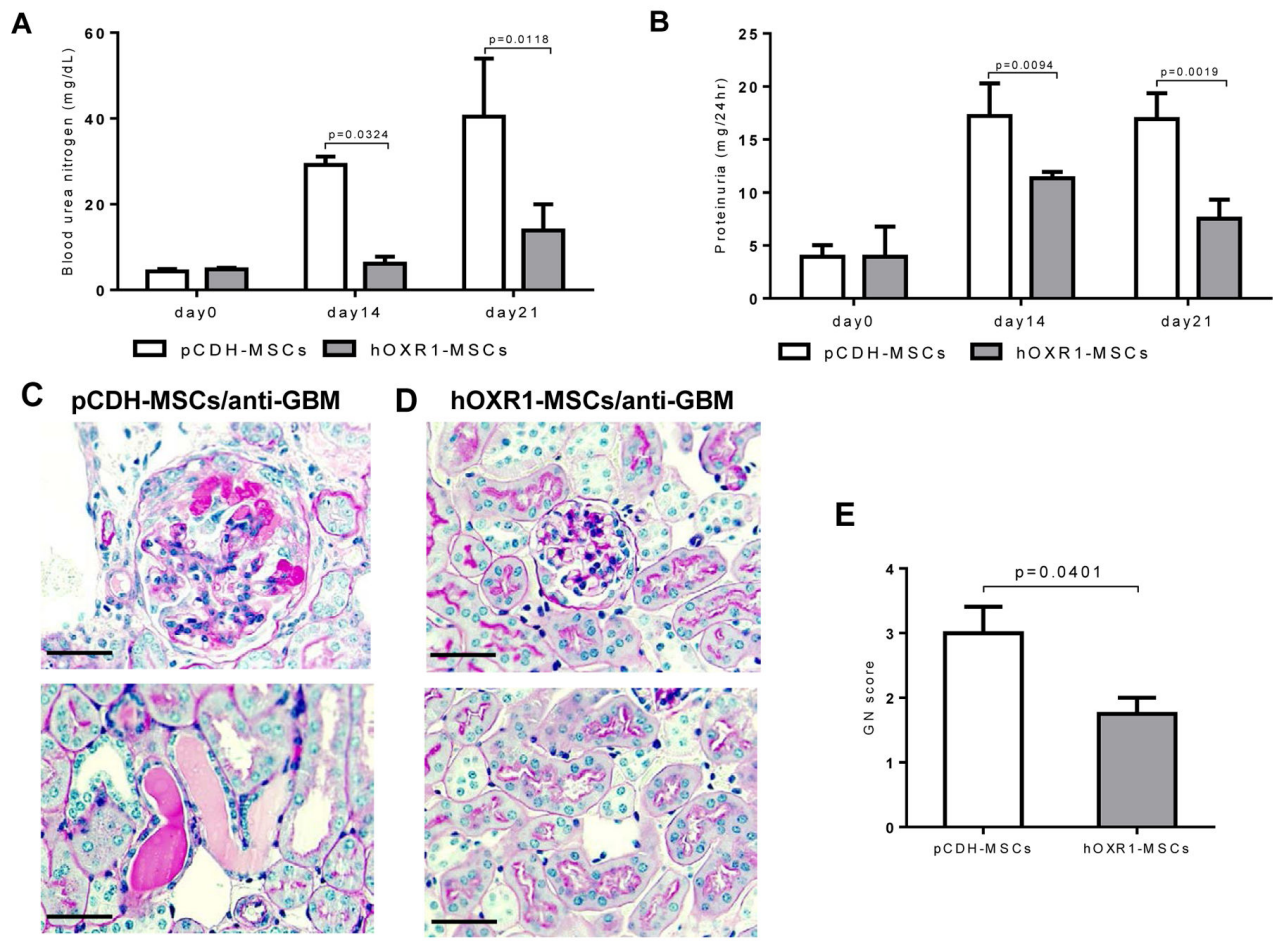
hOXR1-MSCs transplantation suppressed anti-GBM induced nephritis in *129/svj* mice. (A) BUN levels in hOXR1-MSCs and pCDH-MSCs treated anti-GBM challenged 129/svj mice. (B) Twenty-four hour proteinuria levels in hOXR1-MSCs and pCDH-MSCs treated anti-GBM challenged 129/svj mice. (C-D) Representative images of PAS stained renal sections from pCDH-MSCs injected or hOXR1-MSCs injected *129/svj* mice with anti-GBM nephritis. (C) Exemplified is some of the typical light microscopic features in anti-GBM induced nephritis in *129/svj* mice with pCDH-MSCs treatment. (D) hOXR1-MSCs injected mice showed very mild renal histological changes. (E) Renal pathology score of anti-GBM challenged *129/svj* mice treated with pCDH-MSCs, or hOXR1-MSCs. (Original magnification 400X, scale bars=50 μm). Each group had 5 mice.

The effect of hOXR1-MSCs on *129/svj* mice with anti-GBM induced nephritis was evaluated. Proteinuria and BUN were monotered on day 0, day 14, day 21 after hOXR1-MSCs transplantation. BUN levels in the hOXR1-MSCs group were significantly lower compared with the control group on day 14 (6.13 ± 0.82 mg/dL vs. 29.20 ± 0.97 mg/dL) and day 21 (13.89 ± 3.03 mg/dL vs. 40.40 ± 6.78 mg/dL, Figure 5A; p<0.05). Proteinuria level was also significantly lower in hOXR1-MSCs treated mice on day14 (11.33 ± 0.31 mg/24hr vs. 17.21 ± 1.53 mg/24 hr) and day 21 (7.52 ± 0.90 mg/24hr vs. 16.92 ± 1.41 mg/24hr) compared with control mice (Figure 5B; p<0.05). The marked GN noted in the control group was accompanied by significant glomerular proliferation and crescent formation, as depicted in Figure 5C. Histological analysis of kidneys confirmed that *OXR1* gene delivery attenuated histological GN (Figure 5D). *129/svj* mice receiving pCDH-MSCs showed moderate to severe GN with a score of 3.0 ± 0.4, whereas the hOXR1-MSCs injected *129/svj* mice exhibited ameliorated glomerular disease with an average GN score of 1.8 ± 0.3 (Figure 5E; p<0.05). The above findings suggested that the MSCs expressing *hOXR1* were able to suppress immunemediated glomerulonephritis.

### hOXR1-MSCs treatment alleviated lupus nephritis

**Figure 6:**
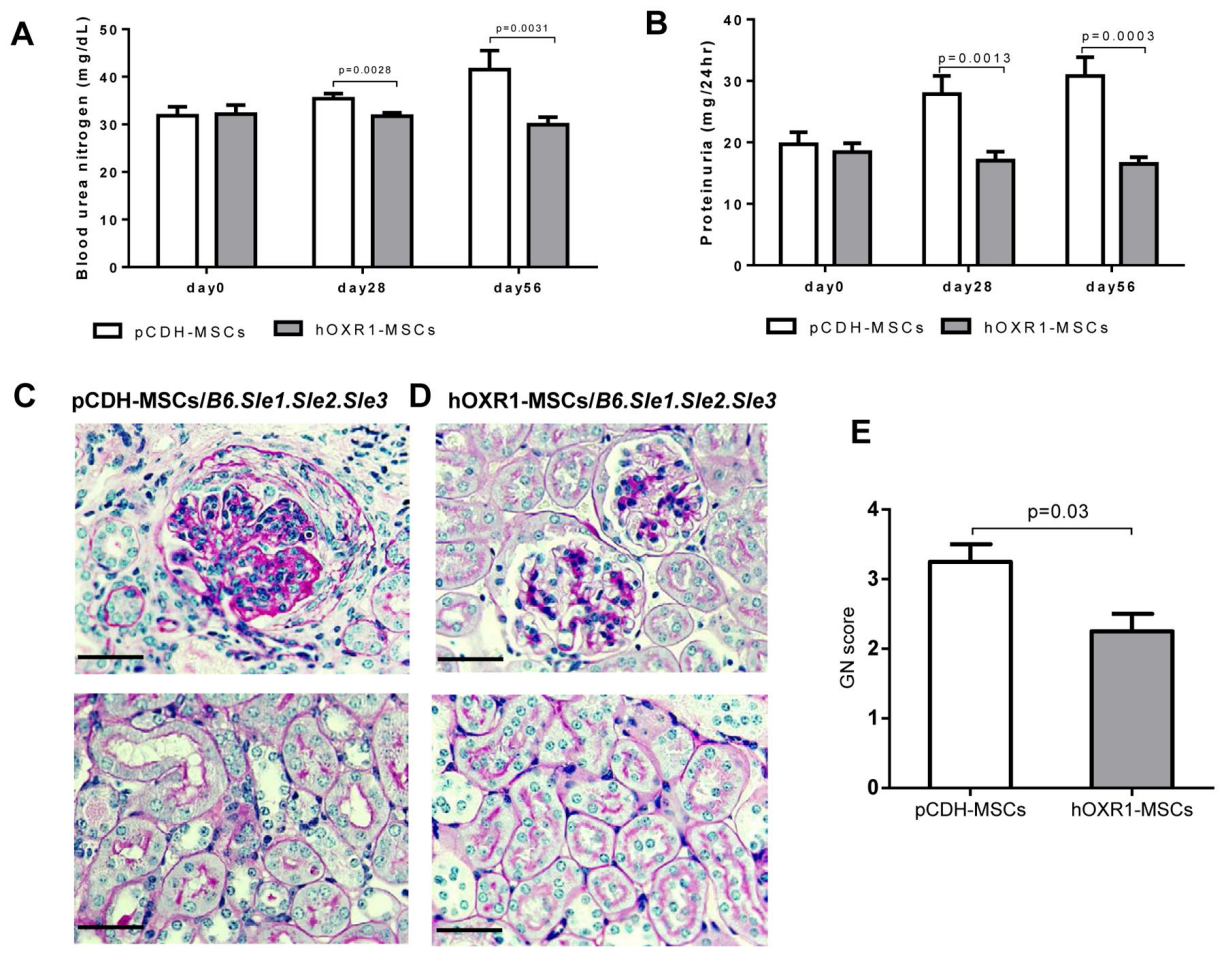
hOXR1-MSCs treatment alleviated lupus nephritis in *B6.Sle1.Sle2.Sle3* mice. (A) BUN (mg/dL). (B) 24 hr proteinuria (mg/24 hr). (C–D) Representative images were PAS stained renal sections from pCDH-MSCs injected, or hOXR1-MSCs injected *B6.Sle1.Sle2.Sle3* mice. (C) Exemplified is some of the light microscopic features seen in lupus nephritis with pCDH-MSCs treatment. (D) Please note the improved renal histological changes after treatment with hOXR1-MSCs. No significant tubulointerstitial changes were observed. (E) Renal pathological score in the two groups of mice. (Original magnification 400X, scale bars=50 μm). Each group had 5 mice.

To determine if hOXR1-MSCs might also be protective against spontaneous lupus nephritis, *B6.Sle1.Sle2.Sle3* mice were injected with hOXR1-MSCs or pCDH-MSCs. BUN and proteinuria were monitored at 4 weeks and 8 weeks after treatment. hOXR1-MSCs treated mice demonstrated significantly reduced levels of BUN after hOXR1-MSCs treatment for 8 week compared with control mice (29.95 ± 0.79 mg/dL vs. 41.50 ± 2.33 mg/dL, as shown in Figure 6A). After 8 week treatment, the proteinuria level was significantly reduced compared to the control mice (16.48 ± 0.55 mg/24 hr vs. 30.77 ± 1.78 mg/24 hr, Figure 6B). Histological analysis confirmed that hOXR1-MSCs treatment significantly attenuated lupus renal pathology (Figure 6C and 6D). pCDH-MSCs treated mice showed severe renal injury (Figure 6C). In contrast, hOXR1-MSCs injected mice exhibited mild to moderate renal injury characterized by mild mesangial proliferation with slightly increased mesangial matrix (Figure 6D). Histological analysis of kidneys confirmed that hOXR1-MSCs treatment attenuated histological GN. The mice receiving pCDH-MSCs showed moderate to severe GN with a score of 3.2 ± 0.3, whereas the hOXR1-MSCs injected mice exhibited ameliorated glomerular disease with a GN score of 2.1 ± 0.3 (Figure 6E; p<0.05).

### hOXR1-MSCs reduced renal cell apoptosis

**Figure 7:**
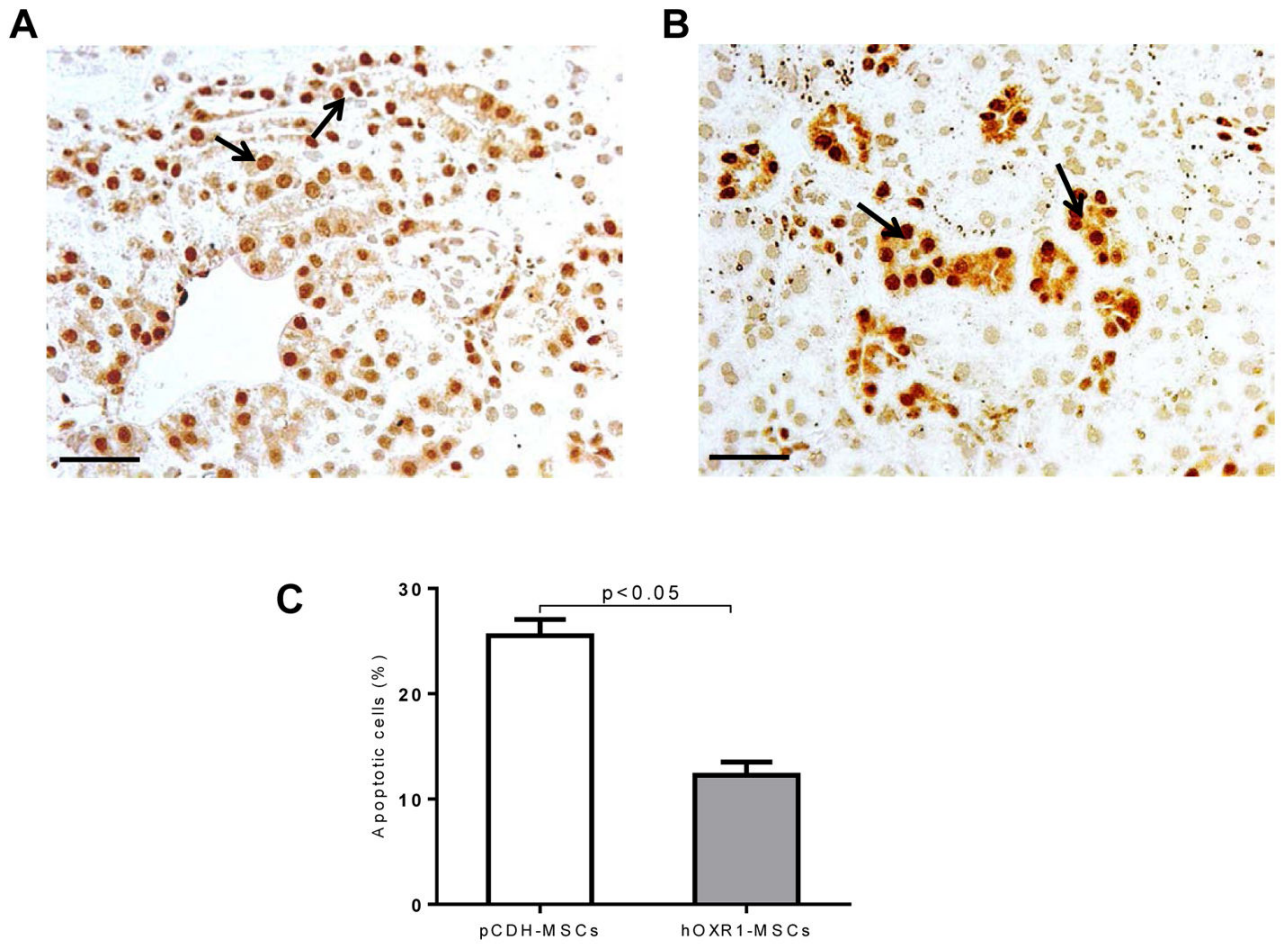
Tunel staining for apoptosis detection in *B6.Sle1.Sle2.Sle3* kidney. (A–C) Detection of apoptotic cells in the kidneys of *B6.Sle1.Sle2.Sle3* mice treated with pCDH-MSCs (A) or hOXR1-MSCs (B) for 8 weeks. Apoptotic cells appeared dark brown (indicated by arrow). (C) Plotted is the quantitative analysis of the percentage of renal apoptotic cells in the 2 groups of mice. (Original magnification 400X, scale bars=50 μm). Each group had 5 mice.

*B6.Sle1.Sle2.Sle3* mice injected with hOXR1-MSCs exhibited significantly reduced apoptotic cells in the kidneys compared with the mice injected with pCDH-MSCs by Tunel staining (12.3 ± 1.3% vs. 22.5 ± 1.6%, p<0.05. Figure 7A–7C).

### hOXR1-MSCs dampened inflammatory cell infiltration in lupus nephritis

**Figure 8:**
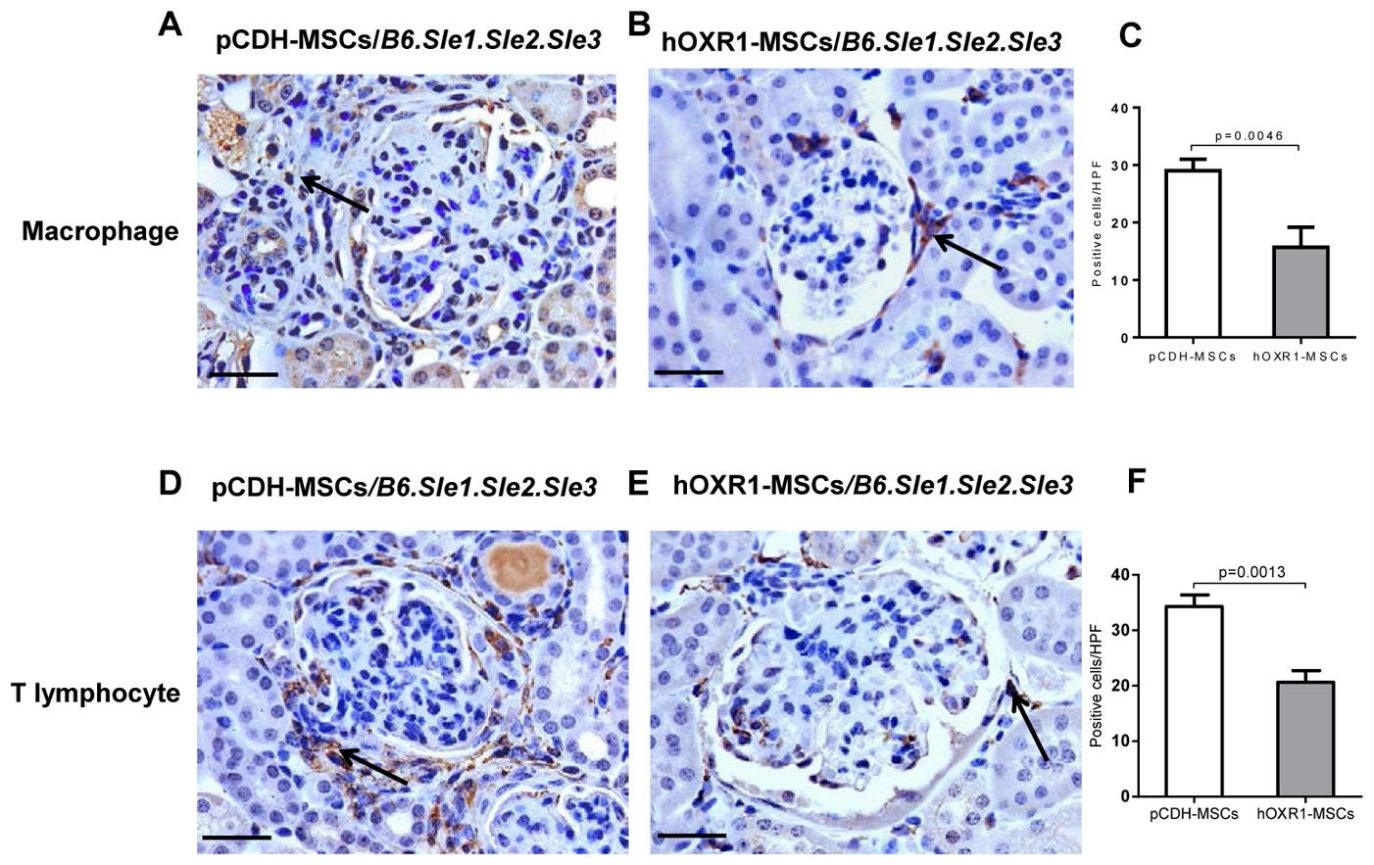
Macrophages and T lymphocyte infiltration into lupus kidneys. (A–B) Iba1 staining for macrophage infiltration into the kidneys of pCDH-MSCs or hOXR1- MSCs treated *B6.Sle1.Sle2.Sle3* mice. (C) Quantitative analysis of Iba1 positive cells in the kidney. The number of Iba1 positive cells in the kidney of hOXR1-MSCs treated mice was significantly less than that in pCDH-MSCs treated mice (p<0.05). n=5 mice per group. (D–E) CD3 staining for T lymphocyte infiltration into the kidneys of pCDH-MSCs or hOXR1-MSCs treated *B6.Sle1.Sle2.Sle3* mice. (F) Quantitative analysis of CD3 positive cells in the kidney. The number of CD3 positive cells in the kidney of hOXR1-MSCs treated mice was significantly less than that in pCDH-MSCs treated mice (p<0.05). (Original magnification 400X, scale bars=50 μm). n=5 mice per group.

We investigated the effect of hOXR1-MSCs transfer on macrophage and T lymphocyte infiltration into the kidneys of *B6.Sle1.Sle2.Sle3* mice. Macrophages were significantly reduced in hOXR1-MSCs administrated mice compared with pCDH-MSCs treated mice, as shown in Figure 8A and 8B. The quantitative analysis of Iba1 positive cells in the kidneys of pCDH-MSCs treated mice and hOXR1-MSCs treated mice is shown in Figure 8C (29 ± 1 vs. 16 ± 2, p<0.05). T lymphocyte infiltration in hOXR1-MSCs treated mice was also reduced compared with pCDH-MSCs treatment (21 ± 1 vs. 34 ± 1; p<0.05), as shown in Figure 8D–8F. hOXR1-MSCs treatment also decreased renal macrophage and T lymphocyte infiltration in anti-GBM challenged *129/svj* mice (data not shown).

### Oxidation associated proteins in kidney regulated by hOXR1- MSCs treatment

**Figure 9:**
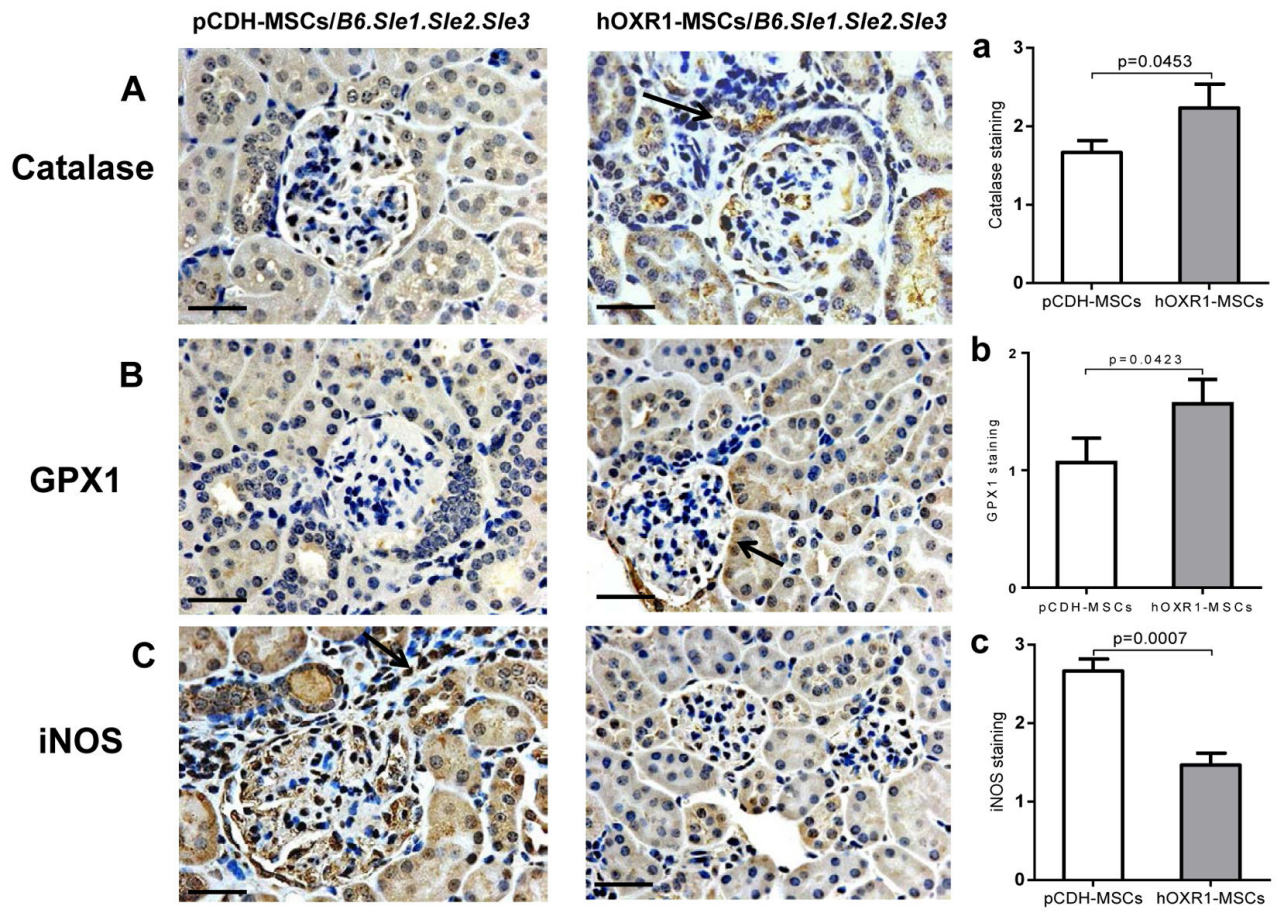
Oxidation associated protein expression in kidney tissue of *B6.Sle1.Sle2.Sle3 mice*. (A) Catalase staining in kidneys of pCDH-MSCs or hOXR1-MSCs treated mice. Semi-quantitative analysis of catalase staining in kidney. The signal of catalase in hOXR1-MSCs treated mice was significantly stronger than that in control mice (p<0.05). n=5 mice per group. (B) GPX1 detection in kidneys of pCDH-MSCs or hOXR1-MSCs treated mice. Semi-quantitative analysis of GPX1 staining in kidney. The signal of GPX1 in hOXR1-MSCs treated mice was significantly stronger than that in control mice (p<0.05). n=5 mice per group. (C) iNOS expression in the kidneys of pCDH-MSCs or hOXR1-MSCs treated mice. Semi-quantitative analysis of iNOS staining in the kidneys. The signal of iNOS in hOXR1-MSCs treated mice was significantly weaker than that in control mice (p<0.05). (Original magnification 400X, scale bars=50 μm). n=5 mice per group.

Catalase, GPX1 and iNOS were monitored in *B6.Sle1.Sle2.Sle3* kidneys by immunohistochemical staining. The staining signal for catalase and GPX1 were stronger kidneys from mice treated with hOXR1-MSCs than that in pCDH-MSCs treated control mice. Representative images were shown in Figure 9A and 9B. Semi-quantitative analysis of catalase and GPX1 staining is shown in Figure 9a and 9b. However, renal iNOS signal was weaker in hOXR1-MSCs treated mice than that in pCDH-MSCs treated control mice (Figure 9C and 9c). We found similar results in *129/svj* mice subjected to anti- GBM challenge (data not shown).

### hOXR1-MSCs transfer also reduced the nitric oxide level in serum and urine

**Figure 10:**
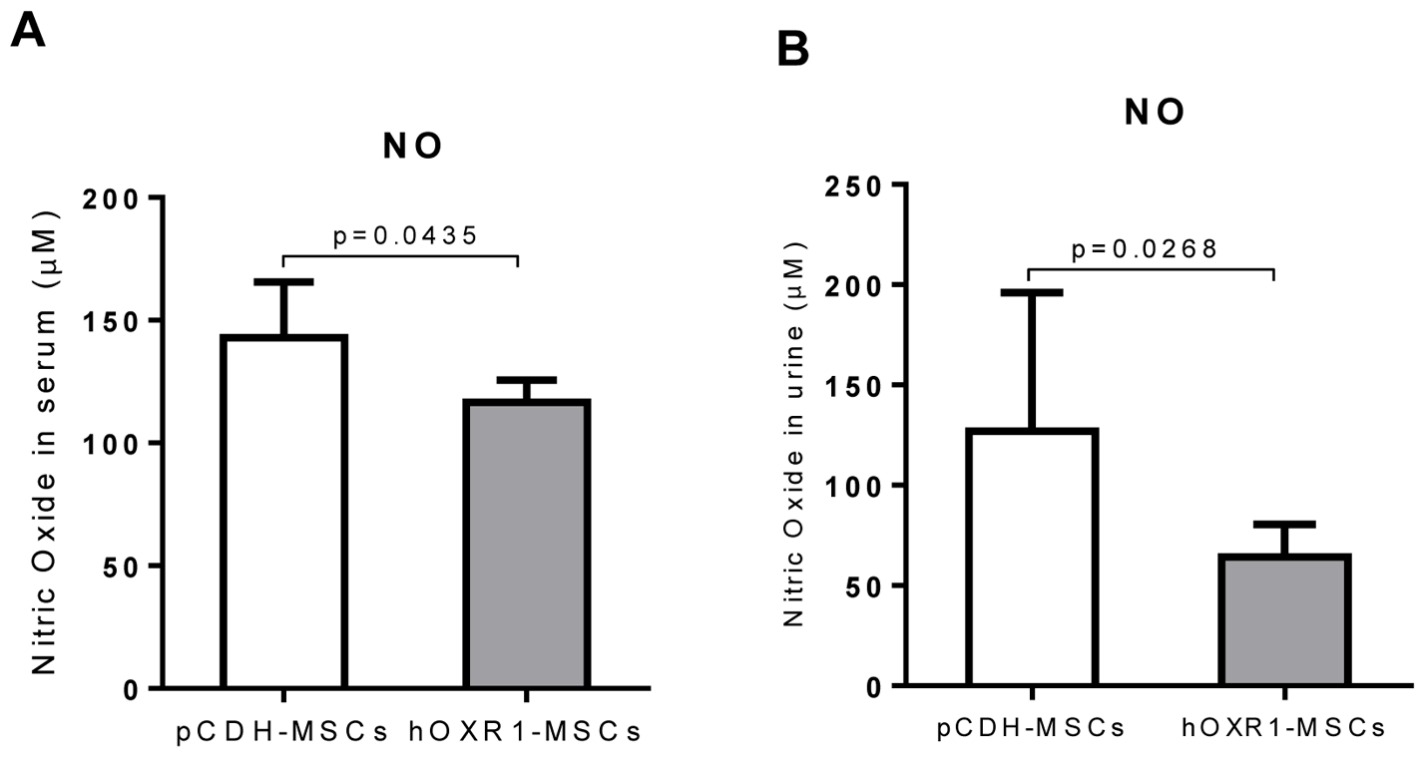
Nitric oxide level in serum and urine of *B6.Sle1.Sle2.Sle3* mice. Nitric oxide levels in the serum of pCDH-MSCs or hOXR1-MSCs treated mice. Nitric oxide was significantly reduced in the serum of hOXR1-MSCs treated mice compared with pCDH-MSCs treated mice (p<0.05). n=5 mice per group. Nitric oxide levels in the urine of pCDH-MSCs or hOXR1-MSCs treated mice. Nitric oxide was significantly lower in the urine of hOXR1-MSCs treated mice compared with pCDH-MSCs treated mice (p<0.05). n=5 mice per group.

In order to evaluate the effect of hOXR1-MSCs on systemic disease activity and oxidative status, we assayed nitric oxide levels in mouse serum and urine. Nitric oxide levels were significantly decreased in the serum and urine of hOXR1-MSCs treated *B6.Sle1.Sle2.Sle3* mice compared with that of pCDH-MSCs treated mice as shown in Figure 10A (116.6 ± 4.1 μM vs. 142.9 ± 10.2 μM, p<0.05) and Figure 10B (64.3 ± 5.7 μM vs. 126.9 ± 26.1 μM, p<0.05).

### hOXR1-MSCs treatment suppressed expression of inflammatory cytokines and up-regulated antioxidants

**Figure 11:**
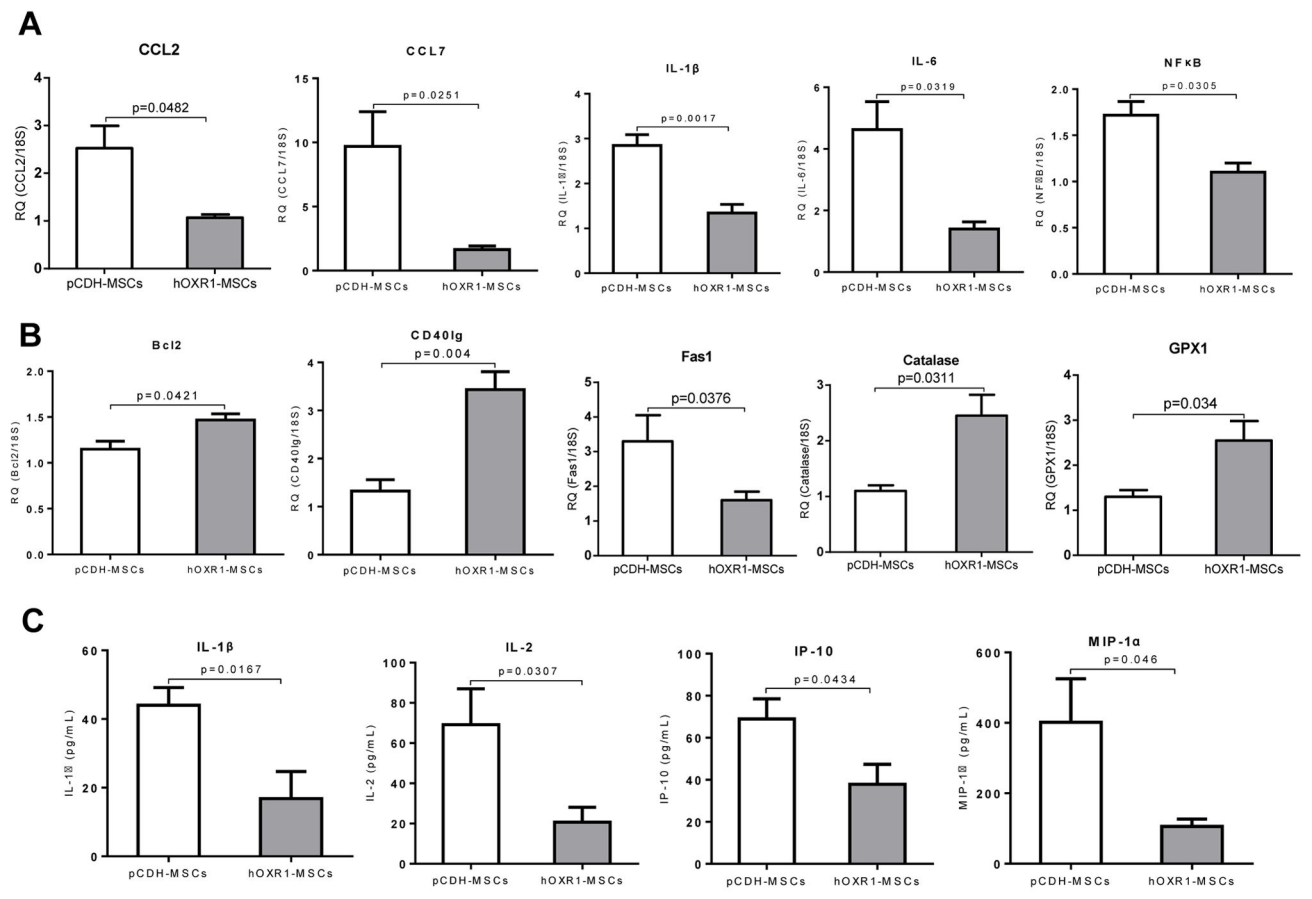
Expression level of inflammatory cytokines and antioxidants in kidneys of *B6.Sle1.Sle2.Sle3* mice after treatment. (A) Differential expression of inflammatory cytokines in the kidney of pCDH-MSCs or hOXR1-MSCs treated mice by QPCR (p<0.05, n=5 in each group). (B) Differential expression of apoptosis-associated genes and antioxidant genes in kidneys of mice treated with pCDH-MSCs or hOXR1-MSCs by QPCR (p<0.05, n=5 in each group). (C) Down-regulation of expression of cytokines in serum of mice treated with hOXR1-MSCs compared with control mice (p<0.05, n=3 in each group).

QPCR analysis of kidney from hOXR1-MSCs treated *B6.Sle1. Sle2.Sle3* mice and pCDH-MSCs treated mice showed that some inflammatory cytokines and chemokines were down-regulated in hOXR1-MSCs treated mice compared with pCDH-MSCs treated mice, such as CCL2, CCL7, IL-1β, IL-6 and NFκB, as shown in Figure 11A. In addition, the expression of apoptosis associated genes, Bcl2 and CD40L was significantly increased in the hOXR1-MSCs treated mice compared with the control mice, but Fas1 was significantly reduced after hOXR1- MSCs treatment (Figure 11B; p<0.05). Other proinflammatory mediators, such as CXCL10, IL-10, MMP2 and TNF, also showed a decreasing trend in hOXR1-MSCs treated mice compared with the control mice (data not shown). More importantly, we found two antioxidants, catalase and GPX1 were significantly up-regulated in hOXR1-MSCs treated mice compared with the control *B6.Sle1.Sle2.Sle3* mice, QPCR results are shown in Figure 11B. We also found that serum IL-1β, MIP-1α, IL-2 and IP-10 were markedly reduced in hOXR1-MSCs injected mice compared with the control mice, as shown in Figure 11C.

## Discussion

Mounting evidences suggests that oxidative stress is increased in systemic lupus erythematosus (SLE), and contributes to immune system dysregulation, abnormal activation and processing of cell-death signals [22,23]. The over-production of reactive oxygen species (ROS) and oxidative free radicals are the primary cause of oxidative stress in SLE and positively correlated with disease activity [17,18,24,25]. Protein oxidation played an important role in the pathogenesis of chronic organ damage in SLE, especially in lupus nephritis [20,26,27]. Generally, cells have a variety of antioxidant systems to defend against free radical attack [26–28]. For instance, superoxide dismutase (SOD) catalyzes the dismutation of the superoxide anion into H_2_O_2_, and H_2_O_2_ can be transformed into H_2_O and O_2_ by catalase, and GPX reduces lipidic or nonlipidic hydroperoxides, as well as H_2_O_2_ while oxidizing glutathione [29,30]. The imbalance of oxidative status is one of the prominent features of SLE, associated with increased plasma MDA and impaired GSH [25,26]. Increased T lymphocyte apoptosis has also been shown to be mediated by decreased intracellular glutathione concentration [31–33]. In SLE, the accumulation of oxidative products could be attributed to the over production of free oxidative radicals or impaired antioxidant activity. Therefore, antioxidant molecules could play important role in combating with oxidative stress.

In this study, we investigated the potential influence of a key antioxidant gene, oxidation resistance 1, on inflammatory damage and oxidative stress in lupus nephritis and anti-GBM induced disease and its therapeutic potential. *OXR1* gene is found in all eukaryote genomes, functions in protection against oxidative damage and its homologs are highly conserved in eukaryotes examined so far [10,15]. The overall sequence identity between human and mouse *OXR1* is over 84%. In this study, we used human *OXR1* instead of mouse OXR1 for two considerations. One is that the human *OXR1* protein has been tested to be able to suppress DNA damage in *Escherichia coli* oxidative repair-deficient mutants [12], and when localized to the mitochondria, was sufficient to prevent oxidative damage in *Saccharomyces cerevisiae* mutants lacking *OXR1* [13–15,34]. The second is that the testing of human OXR1 will make it more feasible to be translated for treatment of human lupus nephritis if proved to be effective in mouse study.

We found *OXR1* expression to be higher in SLE patients with severe LN (WHO class III and IV) than those with mild LN (WHO class II) and control subjects, as well as being higher in spontaneous murine lupus nephritis than in healthy control mice, indicating that oxidative stress may correlate with severity of lupus nephritis. In order to test if the of OXR1 could be a potential therapeutic target for the treatment of oxidative damage in anti-GBM disease and LN, we cloned the human *OXR1* gene and constructed an OXR1-MSC cell line which constantly expressed human OXR1. The *in vitro* cultured OXR1-MSCs retained the properties of MSCs and exhibited enhanced resistance to H_2_O_2_- induced apoptosis. We then tested the *in vivo* effects of OXR1-MSCs in mice suffering from acute anti-GBM disease or spontaneous lupus nephritis, both diseases involved in renal oxidative damage [7,19]. Our data indicated that OXR1-MSCs exhibited significant protective effects on both anti-GBM induced renal injury and spontaneous lupus nephritis, compared with control MSCs. Our previous study has shown that injection of mice with MSCs alone had very limited protective effect on either anti-GBM induced nephritis or lupus nephritis, whereas genetic modified MSCs exhibited significantly better effect [21]. Taken together with this study, our data indicated that genetic modification of MSCs with potential functional genes could significantly improve the renal protective function of MSCs possibly by enhancing its anti-apoptosis and anti-oxidant activity. Furthermore, the expression and secretion of target gene product to the renal tissue by genetic modified MSCs could play important therapeutic role in combating oxidative stress induced inflammation.

We also found that *OXR1* could inhibit renal cell apoptosis compared with control treatment, and the protection correlated with the up-regulation of anti-apoptosis genes Bcl2 and CD40L, and down-regulation of the apoptosis-associated gene Fas1 [35,36]. More interestingly, OXR1 could regulate the expression of antioxidants. Two important detoxification enzymes, catalase and GPX1 were up-regulated in the kidneys of mice with hOXR1-MSCs treatment compared to the control mice. Catalase and superoxide dismutase have been found to be associated with oxidative stress in patients with rheumatoid arthritis and systemic lupus erythematosus [37]. GPXs as a major antioxidant can catalyze the reduction of hydrogen peroxide, organic hydroperoxide, and lipid peroxides by reduced glutathione, thereby protecting cells against oxidative damage [38]. Collectively, these findings indicate that OXR1 resets the oxidative imbalance in lupus.

Literature reports also indicate that NO production is increased in SLE, and serum nitrite and nitrate levels have been reported to correlate with disease activity and damage in SLE [39]. NO also plays a crucial role in T cell dyregulation in SLE [40]. NO impacts the oxidative status of T lymphocyte in lupus [41]. The increased NO production may alter the redox balance by generating peroxynitrite through its reaction with superoxide. In this study, we found serum and urine NO levels in hOXR1-MSCs treated mice to be significantly reduced compared with control mice. Thus, hOXR1-MSCs transfer could inhibit oxidative stress in lupus nephritis through various avenues.

One of the characteristic features of lupus nephritis is leukocyte infiltration. In the present study, we found hOXR1-MSCs transfer into lupus nephritis mice inhibited macrophage and T lymphocyte infiltration into kidneys accompanied by the down-regulation of cytokines associated with cell infiltration, such as monocyte chemotactic protein-1 (MCP-1), CCL7/MCP-3, IL-1β, IL-2, IL-6 and IP-10. MCP-1 is expressed by activated monocyte/macrophages, Th1 cells, and natural killer cells, and it attracts leucocytes and other mediators into sites of inflammation [42]. Others have reported that serum concentrations of MCP-1/CCL2, MIP-1/CCL4, RANTES/CCL5 and IP10/CXCL10 in patients with SLE are significantly higher than healthy controls and that MCP-1/CCL2 was positively associated with disease activity in SL [43,44]. Likelywise, IL-6 is another sensitive marker of disease activity and pathogenesis in SLE [45,46]. Thus, *OXR1* transfer inhibits the levels of several disease associated mediators in SLE.

Collectively, the findings of this study indicate that *OXR1* and oxidative stress are critical players in the pathogenesis and progression of lupus nephritis, in both acute anti-GBM nephritis and chronic lupus nephritis. The anti-apoptosis impact of *OXR1* may have resulted in longer-surviving MSCs, and this may have contributed in part to the beneficial impact on lupus nephritis. More importantly, OXR1-MSC transfer had a significant impact on redox balance, pro-inflammatory cytokine/chemokine production and infiltration of leukocytes into nephritis kidneys. Whether these distinct events are causally related to each other remains to be explored. These findings also raise hope that targeting oxidative stress in lupus nephritis may be beneficial.
